# A novel biomarker of human exposure to *Aedes albopictus* based on the Ag5-3 salivary protein from the tiger mosquito

**DOI:** 10.1186/s13071-025-07118-x

**Published:** 2025-11-19

**Authors:** Maria Greta Dipaola, Eleonora Perugini, Giulia Mancini, Nicolò Gennari, Paola Serini, Giulia Bevivino, Alessio Borean, Fabrizio Lombardo, Marco Pombi, Fabrizio Montarsi, Paolo Gabrieli, Federico Forneris, Bruno Arcà

**Affiliations:** 1 Department of Public Health and Infectious Diseases, Sapienza University of Rome, Rome, Italy; 2 Department of Biology and Biotechnology “L. Spallanzani”, University of Pavia, Pavia, Italy; 3 Department of Transfusion Medicine, San Martino Hospital, Belluno, Italy; 4 Istituto Zooprofilattico Sperimentale Delle Venezie, Legnaro, Italy; 5 Department of Biosciences, University of Milan, Milan, Italy

**Keywords:** *Aedes albopictus*, Ag5-3, 34k2, Salivary protein, Host exposure, Serological marker, Vector control, Human-vector contact

## Abstract

**Background:**

Mosquito-borne arboviral diseases represent a growing threat and serious worldwide concern for public health authorities. Host immunoglobulin G (IgG) responses to mosquito salivary antigens emerged as a useful additional tool to evaluate human–vector contact, which is crucial for transmission risk assessment and planning vector control interventions. We previously reported that IgG responses to the *Aedes albopictus* 34k2 salivary protein (al34k2) are suitable, although with some limitations, to reveal variation of human exposure to the tiger mosquito. In this study we evaluated the *Ae. albopictus* Ag5-3 (alAg5-3), an Antigen 5 family member specifically and abundantly expressed in the saliva of adult females.

**Methods:**

IgG responses to recombinant alAg5-3, as well as to a combination of alAg5-3 and al34k2, were measured in a set of sera previously collected from healthy human blood donors before and after the summer season of exposure to mosquito bites. Surveys were conducted in two districts of Northeast Italy, Padua and Belluno, with different density and history of colonization by the tiger mosquito *Ae. albopictus*.

**Results:**

A preliminary pilot study, performed on a small subset of individuals from Padua, indicated that alAg5-3 was more immunogenic than al34k2 and may be suitable to detect variations of exposure to *Ae. albopictus*. Analysis of the whole set of 523 sera showed that anti-alAg5-3 IgG levels significantly increased, in both study areas, after the summer period of high mosquito density. However, differences between the two study sites were only found when a mixture of the two antigens, alAg5-3 and al34k2, was used.

**Conclusions:**

IgG responses to alAg5-3 represent a novel appropriate marker to evaluate seasonal variation of human exposure to *Ae. albopictus* and, because of its higher sensitivity, it appears preferable to al34k2, especially for longitudinal studies in conditions of low-to-moderate mosquito density. However, the combination of both antigens may be a better surrogate of *Ae. albopictus* saliva since it allows the detection of both temporal and spatial variations of exposure to *Ae. albopictus* bites. The high conservation of the Ag5-3 protein among *Aedes* species suggests it may be exploited to also reveal exposure to *Aedes aegypti* and perhaps to other *Aedes* species.

**Graphical Abstract:**

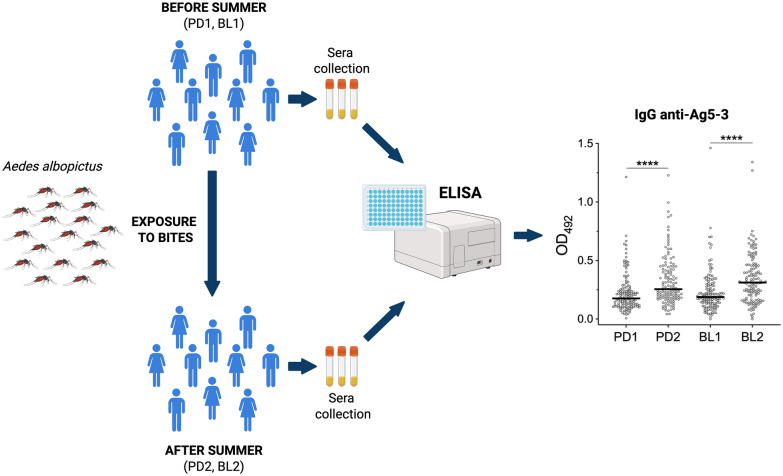

**Supplementary Information:**

The online version contains supplementary material available at 10.1186/s13071-025-07118-x.

## Background

Vector-borne diseases, according to the World Health Organization (WHO), account for over 17% of all infectious diseases worldwide and cause at least 700 thousand deaths annually. Mosquitoes of the genus *Anopheles*, which transmit *Plasmodium* parasites, and of the genus *Aedes*, which transmit arboviruses of great relevance to human health (as dengue, Zika, chikungunya, or yellow fever viruses) are responsible for more than 90% of these deaths [1]. More than 5.6 billion people are estimated to live in areas suitable for dengue, chikungunya, and Zika [2], and these diseases represent a growing and challenging threat for public health [3]. In fact, over the past decades, these major arboviruses not only caused severe outbreaks in tropical or subtropical endemic areas but also emerged and eventually spread into new regions where they were previously absent. In this context, the recent severe outbreaks of Zika and dengue in Latin America [4, 5] and the several cases of local transmission in Mediterranean Europe and Southern USA [6–8] sound as an alarm bell for public health authorities.

The yellow fever mosquito *Aedes aegypti* and the Asian tiger mosquito *Aedes albopictus* are by far the most important arboviral disease vectors, with *Ae. aegypti* certainly playing the most prominent role. However, the ecological plasticity exhibited by *Ae. albopictus*, which allows for adaptation to temperate climates [9], and its competence in the transmission of several arboviruses [10], make the tiger mosquito a relevant epidemic driver, especially in epidemiological settings where *Ae. aegypti* is absent or present to a limited extent. This is clearly testified by the large outbreaks caused by chikungunya and dengue viruses in the Réunion Island [11, 12], and by the several cases of autochthonous arboviral transmission in the European continent [6, 7, 13, 14]. These two *Aedes* vectors, owing to several factors (e.g., global warming with associated climate changes and human activities including international commerce/travel and ecological disruptions) have progressively expanded their distribution into new tropical, subtropical, and temperate areas, with implications for arboviral transmission and spreading [15–17]. As far as the European continent is concerned, *Ae. albopictus* is currently established or present in at least 20 countries with a northward expansion up to Southern Sweden [18], whereas *Ae. aegypti* is now present at the borders of continental Europe (Cyprus, Madeira, Gran Canaria, and around the Black Sea [19]), with a concrete risk of introduction into the Mediterranean area. Moreover, the invasive species *Aedes koreicus* and *Aedes japonicus* are also well established in multiple European countries [20, 21], increasing the concern for mosquito-borne diseases in Europe [13, 22, 23], even though their health importance and vector capacity has not yet been clearly evaluated.

No specific antiviral drugs are currently available to treat these arboviral diseases. A safe, effective, and widely tested vaccine against yellow fever has been available for some time [24], and vaccines for dengue (Qdenga^®^, TAK-003) and chikungunya (IXCHIQ) have been more recently developed; nevertheless, their use is still limited and/or follows specific recommendations [25, 26]. Novel approaches to reduce the burden of arboviral diseases, including the use of symbionts and of genetically modified mosquitoes, are currently being explored, with the use of *Wolbachia* for vector population suppression or replacement being the most advanced and promising strategy thus far [27–30]. Therefore, vector monitoring and control, along with prevention of mosquito bites, still represent the main strategies for the containment of arboviral diseases transmission. For *Aedes* mosquitoes, the evaluation of human–vector contact is traditionally based on the assessment of vector mosquito density (by ovitraps, larval/pupal indices, adult traps) and/or of biting index (by human landing catches, HLC) [31]. However, these entomological methods can be costly and labor-intensive, may be difficult to implement in some epidemiological setting (e.g., low vector density, logistic constraints), or may raise ethical concerns (HLC); moreover, in most cases they provide only an indirect and approximate estimation of human–vector contact at the community level. A more recently emerged methodology for the assessment of human exposure to disease vectors is based on the measure of circulating antibodies against the salivary proteins that hematophagous arthropods inject into their hosts during blood feeding [32]. Such an additional complimentary tool may be very helpful since it provides a direct measure of human exposure (not only at the community but also at the individual level), can be implemented in settings in which the estimation of other entomological outcomes is not applicable, and may be used to obtain information on both pathogen circulation and host exposure to vectors by simple serological measurements. Moreover, it may represent a very useful tool to assess the efficiency of vector control interventions. In the case of mosquitoes, this was initially shown for both anophelines and culicines by measuring IgG antisaliva or antisalivary gland protein extracts (SGE) [33–35]. Afterwards, transcriptomic and proteomic studies shed light on the complexity of blood feeding insect saliva [36], leading to the identification of *Anopheles*- and *Aedes*-specific salivary proteins [37]; these genus-specific salivary proteins represent ideal candidates for the development of immunoassays based on individual recombinant or synthetic salivary antigens to specifically assess human exposure to malaria or to arboviral vectors, respectively. Importantly, the use of single genus-specific mosquito salivary proteins is also expected to improve reproducibility and to overcome potential cross-reactivity owing to the fact that mosquito saliva is a complex cocktail of at least 100–150 salivary proteins and that some of them are widely spread among blood feeding insects [32, 36, 37].

A convincing proof of concept has been provided for malaria vectors, with most studies using the anopheline-specific salivary protein gSG6 from *Anopheles gambiae* or the derived gSG6-P1 peptide [38]. In fact, they have been widely validated as markers of human exposure to Afrotropical malaria vectors in different epidemiological setting [39–46], with indications of their possible use for Asian [47, 48] and perhaps Polynesian [49, 50] malaria vectors. Efforts toward the development of similar tools for *Aedes* mosquitoes have been obviously focused, considering their crucial role in arboviral transmission, on *Ae. aegypti* and *Ae. albopictus*. The first antigen used with encouraging results in different epidemiological settings (Benin, Cote d’Ivoire, Lao PDR) was the Nterm-34 kDa peptide, which is designed on the amino-terminal region of the *Ae. aegypti* 34k1 salivary protein [51–54]. This same peptide was also used with some success to evaluate exposure to *Ae. albopictus* at La Réunion Island [55], although it may lack sensitivity in assessing exposure to the tiger mosquito because it only partially matches the N-terminal region of the *Ae. albopictus* 34k1 protein (12/19 identical residues, with three amino acids gap). More recently, Chea and collaborators, using a set of sera from a longitudinal cohort study conducted on Cambodian children, screened a panel of 18 *Ae. aegypti* salivary proteins and showed that IgG responses to the *Ae. aegypti* D7L1 and D7L2 salivary proteins were a highly sensitive marker of exposure to the yellow fever mosquito [56].

Toward the development of an effective serological marker to evaluate human exposure to the highly invasive tiger mosquito, we previously tested the suitability of the *Ae. albopictus* salivary protein 34k2 (al34k2), also named LIPS-2 because of its physiological role in probing and feeding by *Aedes* mosquitoes [57]. We found that anti-al34k2 IgG responses could efficiently reveal variation of human exposure to *Ae. albopictus* in areas of high tiger mosquito density but had limited sensitivity in conditions of low or moderate density [58–60]. In this study we measured IgG responses to the *Ae. albopictus* salivary protein Ag5-3 (alAg5-3) The Ag5-3 salivary protein of *Ae. albopictus* (GenBank 56417472/AAV90677 or AY826105; UniProt Q5MIV5) is an Antigen 5 family member that is abundantly and specifically expressed in adult female salivary glands [61]. Other three members of this multigene family, named Ag5-1 (GenBank 56417468/AAV90675 or AY826103, UniProt Q5MIV7), Ag5-2 (GenBank 56417470/AAV90676 or AY826104, UniProt Q5MIV6), and Ag5-4 (GenBank 56417516/AAV90699 or AY826127, UniProt A0A182GL09), were found expressed in the salivary glands of the tiger mosquito, but only Ag5-2 shared with Ag5-3 the same tissue- and sex-specific expression pattern [61]. The presence of four salivary Ag5 proteins in *Ae. albopictus* is likely the result of gene duplication followed by divergent evolution, which is common among salivary proteins of blood feeding arthropods [36]. Notably, Ag5-3 shares only a 31–43% amino acid identity with the other three members of the family. The *Ae. albopictus* Ag5-3, whose predicted mature form is 235 amino acids in length, was previously found to be immunogenic to humans in an immunoproteomic study [62]. Moreover, more recently, individuals with hypersensitive reactions to mosquito bites were found to carry IgE targeting alAg5-3 (also named Aed al 13 according to the allergen nomenclature) [63]. For these reasons the alAg5-3 protein was selected to evaluate its suitability as a marker of human exposure to *Ae. albopictus*. To this end, we used sera collected from healthy human blood donors before and after the summer season of higher exposure to mosquito bites. Surveys were carried out in two areas with different density and history of colonization by the tiger mosquito, Padua and Belluno (Northeast Italy). Therefore, these cohorts were expected to allow for the evaluation of both temporal and spatial variation of human exposure to the tiger mosquito. Notably, the same set of sera was previously used to analyze IgG responses to al34k2 and to *Ae. albopictus* salivary gland protein extracts (alSGE) [58], which offered the opportunity to make some interesting comparisons.

## Methods

### Study areas and sera collection

The study was carried out in 2017 in the cities of Padua and Belluno, both located in the Veneto region in northeastern Italy. Padua, located in a plain area (27 m a.s.l.), is one of the first European cities colonized by the tiger mosquito, with the first finding dating back to 1991 [64]. After its introduction, the tiger mosquito quickly became the most abundant *Aedes* species in the urban area and progressively spread to the entire Veneto region. Belluno, located at 389 m a.s.l. and surrounded by mountains, was colonized by *Ae. albopictus* around 20 years later, in 2012 [65]. In the same period another exotic mosquito species, *Aedes koreicus*, was also reported in the Belluno area [66]; however, according to entomological data collected in the years 2014–2015, that is shortly before the sera collection for this study, *Ae. albopictus* was the prevalent mosquito species in Belluno (57%), followed by *Culex pipiens* (32.1%) and *Ae. koreicus* (9.2%) [67]. Considering the time of colonization and available entomological data, Padua and Belluno were selected as sites with high and low-to-moderate exposure to the tiger mosquito, respectively. A map of the study sites was previously published as Supplementary material [58] and can be easily downloaded at the following link (https://www.frontiersin.org/journals/cellular-and-infection10.3389/fcimb.2020.00377/full#supplementary-material).

Sera were collected among adult blood donors at the immune transfusion centers of Padua (Padua University Hospital) and Belluno (San Martino Hospital). A first collection was done in May 2017 (Padua, PD1 *n* = 130; Belluno, BL1 *n* = 130), that is after a period of approximately 4–5 months of no significant exposure to *Ae. albopictus* bites. The second collection was done from mid-September to end of November 2017 (Padua, PD2 *n* = 132; Belluno, BL2 *n* = 131), after the summer period of high mosquito density. A subset of individuals from Padua (*n* = 69) and Belluno (*n* = 97) participated in both surveys. Volunteers participating to the study were also asked to fill a short questionnaire on travel abroad in the period preceding blood donation as well as on timing, perception of intensity, and skin reactions to mosquito bites. A more detailed description of study areas, sera collection, and questionnaire has been previously provided [58]. A subsample of 31 individuals from Padua was used to compare, in a pilot experiment, IgG responses to al34K2 and alAg5-3; these 31 individuals were randomly selected among those (*n* = 69) for which sera were obtained both before (PD1) and after (PD2) the summer season of high mosquito density. The entire set of 523 sera described above was employed to measure IgG responses to the alAg5-3 antigen alone and to a combination of the two antigens (alAg5-3 and al34k2).

### Entomological monitoring

In parallel to sera collection, entomological surveys were carried out in Padua and Belluno, as previously reported and described in detail by Buezo Montero and collaborators [58]. These two study areas, as also mentioned above, were identified as districts with “high” (Padua) and “low-to-moderate” (Belluno) tiger mosquito density according to: (i) time and history of colonization and establishment in the two sites; (ii) published entomological data [68]; (iii) annual entomological monitoring in the years preceding sera collection (F. Montarsi, unpublished observations). The surveys were conducted in the periods May–July and August-October 2017, using oviposition standard traps, which is the most common type of trap for monitoring *Ae. albopictus* [69, 70]. The mean number of eggs per positive ovitrap and the percentage of positive ovitraps were calculated to estimate the seasonal mosquito density. To simplify the correlation between entomological and serological data, a table summarizing the ovitraps data from Buezo Montero et al*.* [58] is reported as Supplementary material (Supplementary Table S1).

### Evaluation of IgG responses to the Aedes albopictus alAg5-3 and al34k2 salivary proteins

The recombinant salivary proteins alAg5-3 and al34k2 from the tiger mosquito were expressed and purified as previously described [60, 63]. Enzyme-linked immunosorbent assays (ELISA) were performed using flat-bottom 96-well plates (Nunc MaxiSorp, 442404). According to the antigen(s) tested in the different experiments, plates were coated with 50 µl of coating buffer (15 mM Na_2_CO_3_, 35 mM NaHCO_3_, 3 mM NaN_3,_ pH 9.6) containing alAg5-3 alone (5 µg/ml), al34k2 alone (5 µg/ml) or alAg5-3 plus al34k2 (5 µg/ml each), sealed with microplate sealing sheets (Corning 3095) and incubated overnight at 4 °C. After washing, wells were incubated for 3 h at room temperature (RT) with blocking buffer [150 μl, 1% w/v skimmed dry milk in PBST (PBS, 0.05% Tween 20)], washed again, and then incubated overnight at 4 °C with 50 μl of serum diluted 1: 50 in blocking buffer. After washing, plates were incubated (3 h, RT) with 100 μl of polyclonal rabbit anti-human IgG/horseradish peroxidase (HRP) antibody (Dako P0214) diluted 1:5000. After washing, the colorimetric development was carried out (15 min, 25 °C in the dark) with 100 μl of o-phenylenediamine dihydrochloride (OPD, Sigma P8287). The reaction was terminated by adding 25 μl of 2 M H_2_SO_4_ and the optical density at 492 nm (OD_492_) was determined using a Biotek Synergy HT microplate reader. Washes always consisted of a first washing with PBST followed by three additional washings with distilled water.

### Normalization and data analysis

All samples were analyzed in duplicate with the antigen and once without antigen. A cut-off threshold for seropositivity could not be established because obtaining reliable unexposed controls was not feasible, both because of the wide distribution of *Ae. albopictus* in Italy and of mobility of people from noncolonized to colonized areas (e.g., working reasons or vacations), which is difficult to predict while planning sera collection or to consider afterwards. For this reason, we only performed comparisons of IgG levels, which were calculated as the mean OD_492_ value with antigen minus the OD_492_ value without antigen. Samples with a coefficient of variation between duplicates > 20% were retested or not included in the analysis. To minimize the bias, each experiment included samples from both collection sites (Padua and Belluno) and periods (before and after the summer season). To control for intra- and inter-assay variation, each plate included a standard curve made by three-fold dilution series (1:2-1:4374, in duplicate) of a pool of sera identified as hyperimmune to *Ae. albopictus* saliva in a previous study [58]. Coating conditions for the standard curves were identical to those used for the analysis of individual sera (i.e., with alAg5-3 alone or with alAg5-3 plus al34k2 according to the experiment). Optical density (OD) values from each plate were normalized according to the standard curve of that specific plate and then included in the final database. More specifically, titration curves and the Excel software with a three variable sigmoid model and the Solver add-in tool were used for data normalization, as previously described by Corran and collaborators [71]. Samples did not pass any normality or lognormality tests employed (D’Agostino and Pearson, Anderson–Darling, Shapiro–Wilk, Kolmogorov–Smirnov; alpha = 0.05) and, therefore, the nonparametric Mann–Whitney *U* test and Wilcoxon matched-pairs signed rank test were used for the pairwise comparisons among independent and paired groups, respectively. Graph preparation and statistical analyses were performed using the Prism 10.0 GraphPad Software (San Diego, CA, USA). Results of statistical tests are indicated in the figures included in the manuscript as follows (ns, nonsignificant; *, *P* < 0.05; **, *P* < 0.01; ***, *P* < 0.001; ****, *P* < 0.0001).

### Identification of putative orthologs of al34k2 and alAg5-3 in *Aedes koreicus*

The *Ae. koreicus* genome assembly Akor_1.1 [72], available as reference genome at the NCBI website (https://www.ncbi.nlm.nih.gov/datasets/genome/GCA_024533555.2/), was searched by tblastn using the *Ae. albopictus* al34k2 (UniProt Q5MIU2) and alAg5-3 (UniProt Q5MIV5) salivary proteins as queries. Contigs carrying the putative *Ae. koreicus* orthologs were identified (JAHHFK020000192.1 for 34k2, JAHHFK020004975.1 for Ag5-3) and coding regions plus some flanking sequences were extracted and used for annotation using the Artemis tool [73]. The amino acid sequence of the putative ko34k2 and koAg5-3 were reconstructed taking into account the tblastn alignments and manually verifying, in the case of koAg5-3, the presence of the GT and AG conserved dinucleotides at the intron donor and acceptor sites. Percentage of identities and similarities between Ag5-3 and 34k2 salivary proteins from *Ae. albopictus*, *Ae. aegypti*, and *Ae. koreicus* were determined by blast, aligning the predicted mature proteins. This information has been included as Supplementary material (Supplementary Figs. S4 and S5).

## Results

### Analysis of IgG responses to alAg5-3 before and after the summer season in the study areas

**Fig. 1:**
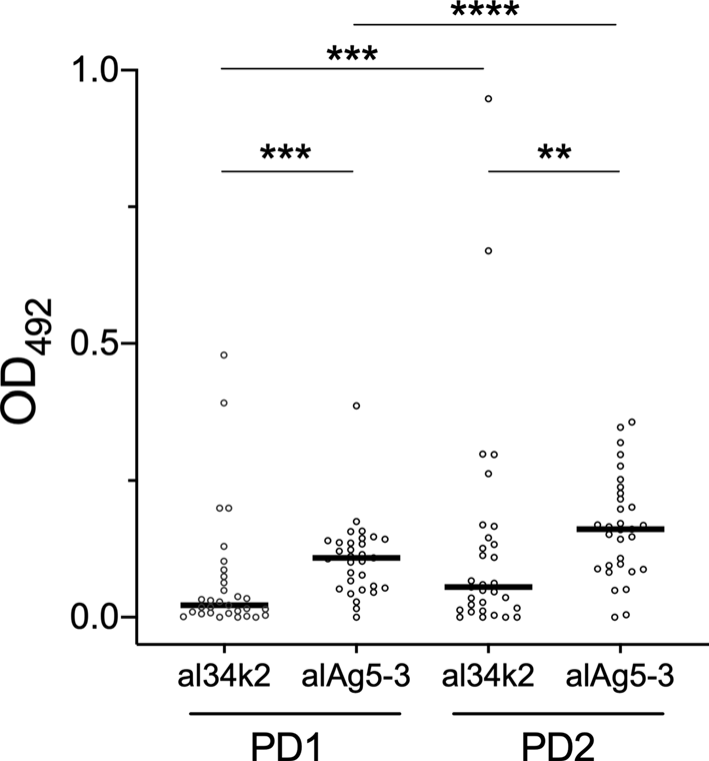
Comparison of immunoglobulin G (IgG) responses to al34k2 and alAg5-3 in a subset of individuals from Padua. Scatter plot of IgG responses to the *Ae. albopictus* recombinant salivary proteins al34k2 and alAg5-3 in 31 randomly selected subjects from Padua who participated to both surveys. IgG levels are expressed as optical density (OD) values. Dots mark individual values, and horizontal bars represent median values. The Wilcoxon matched pairs signed rank test was used for the pairwise comparisons between the two surveys (PD1 versus PD2); the Mann–Whitney *U* test was used to compare IgG responses to al34k2 and alAg5-3 in each of the two surveys, PD1 and PD2

As a first preliminary step, to assess alAg5-3 immunogenicity and verify its possible use for the detection of seasonal variations of human exposure to *Ae. albopictus*, we compared IgG responses to recombinant alAg5-3 in a small subset of 31 subjects randomly selected among the 69 individuals from Padua for which sera were obtained both before (PD1) and after (PD2) the summer season of high mosquito density. We also included in this pilot experiment the *Ae. albopictus* al34k2 salivary protein, which had been already analyzed in previous studies [58–60]; therefore, also anti-al34k2 IgG responses were measured in parallel in the same groups of individuals. As expected from previous observations [58], a significant increase of anti-al34k2 IgG levels (*P* = 0.0002) was observed after the summer period of exposure to mosquito bites (PD1 versus PD2, Fig. 1). A similar result was obtained with alAg5-3 (*P* < 0.0001), with a slightly higher difference of the median values between the PD1 and PD2 groups (0.033 for al34k2 and 0.0525 for alAg5-3).

Interestingly, IgG responses to alAg5-3 were significantly higher than corresponding responses to al34k2 both in the first (PD1, *P* = 0.0002) and in the second (PD2, *P* = 0.0033) survey, pointing to the higher immunogenicity of the alAg5-3 antigen and suggesting it may be more sensitive than al34k2 in detecting variation of exposure to *Ae. albopictus*.

**Fig. 2:**
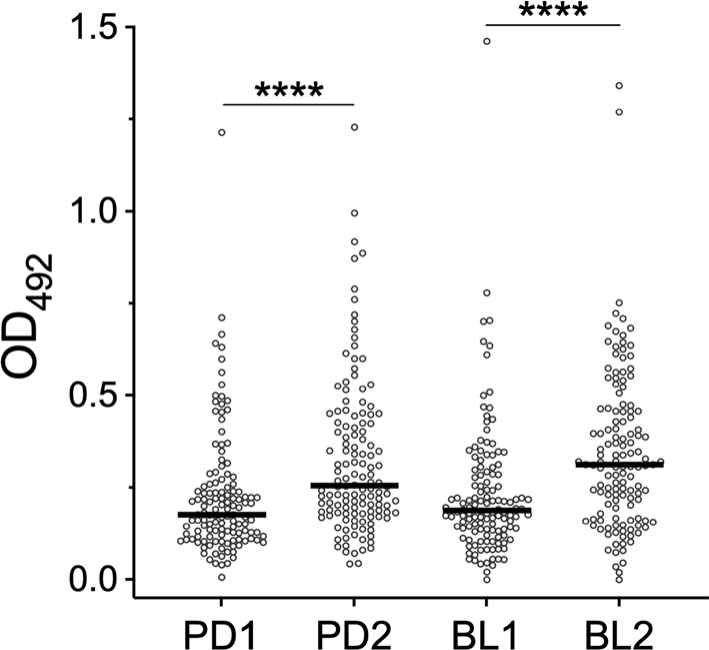
IgG responses to the *Ae. albopictus* alAg5-3. Anti-alAg5-3 IgG levels in participants in the four different surveys (PD1, *n* = 130; PD2, *n* = 132; BL1, *n* = 130; BL2, *n* = 131). IgG levels are expressed as OD values. Dots mark individual values, and horizontal bars represent median values. The Mann Whitney *U* test was used for the pairwise comparisons

The encouraging results obtained in this pilot experiment suggested to extend the analysis to the entire set of 523 sera collected in Padua and Belluno before (PD1 and BL1) and after (PD2 and BL2) the summer season of high mosquito density. As shown in Fig. 2, a highly significant increase of anti-alAg5-3 IgG levels was observed after the summer period of natural exposure to mosquito bites both in Padua and in Belluno (*P* < 0.0001), confirming and extending the observations previously made in the preliminary analysis. Notably, no significant differences were found in IgG responses to alAg5-3 between the two study sites, which were originally identified as areas of “high” (Padua) and “low-to-moderate” (Belluno) *Ae. albopictus* density.

### IgG responses to alAg5-3 according to age

**Fig. 3:**
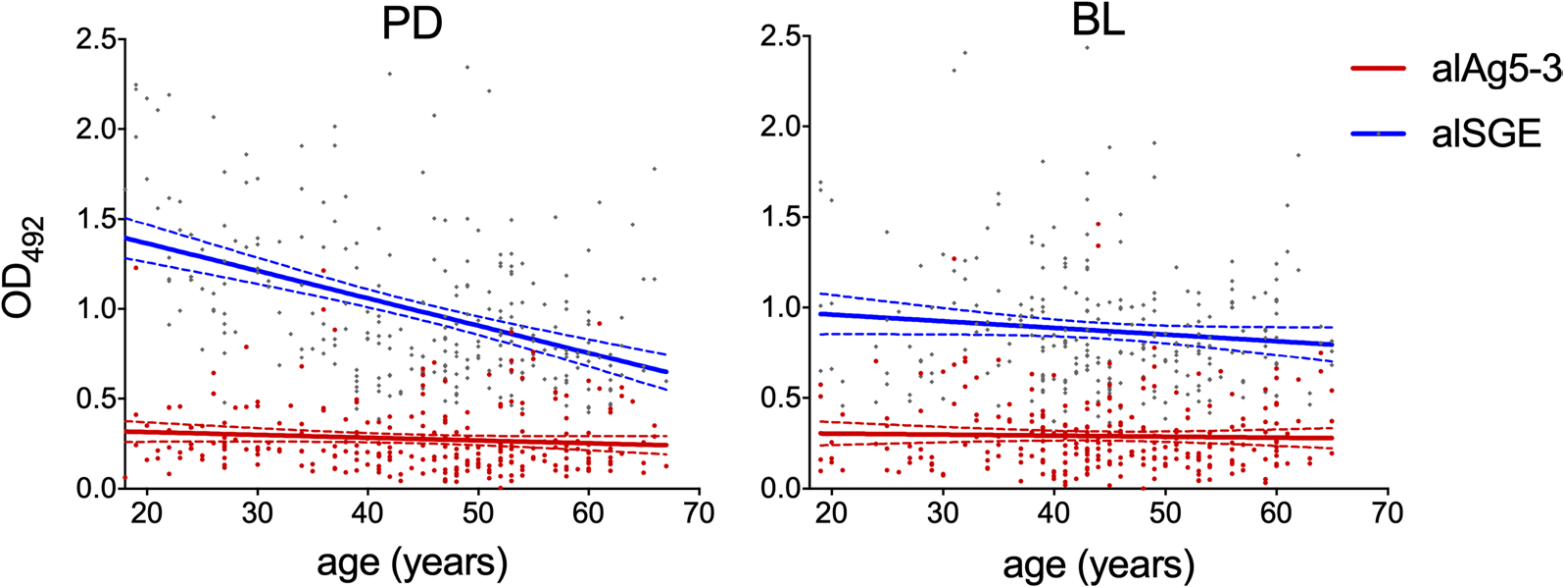
IgG responses to the *Ae. albopictus* alAg5-3 according to age. Scatter plot of IgG responses to alAg5-3 (red) as a function of age in Padua (PD, *n* = 262; left panel) and Belluno (BL, *n* = 261; right panel). Responses to alSGE (blue), as determined in a previous study [58], are shown for comparison. IgG levels are expressed as OD values and dots mark individual values. Best-fit lines (solid lines) and confidence interval bands (dashed lines) are shown. Spearman correlation: PD-alAg5-3 (*r* = −0.1225, *P* = 0.0481), PD-alSGE (*r* = −0.3956, *P* < 0.0001), BL-alAg5-3 (*r* = −0.0207, *P* = 0.7389), BL-alSGE (*r* = −0.0715, *P* = 0.2495)

IgG antibody responses to mosquito saliva and to individual salivary antigens may decrease with age, most likely because of the development of immune tolerance leading to natural desensitization, which may occur especially in conditions of high and persistent exposure to mosquito bites [74–77]. Age-variation of IgG responses to saliva and to individual salivary antigens from *Anopheles* and *Aedes* species has been previously reported [33, 34, 43, 58, 78] and may be antigen-specific as shown for the *An. gambiae* salivary proteins gSG6 and cE5 [44]. For this reason, we also analyzed IgG responses to alAg5-3 according to age. Correlation analyses yielded negative Spearman’s correlation coefficients in most cases, but some weak statistical significance (*P* = 0.0338) was only found for the PD1 survey (Supplementary Fig. S1). The analysis for the two study sites is reported in Fig. 3, where the responses to alSGE, as previously determined [58], are shown for comparison.

**Fig. 4:**
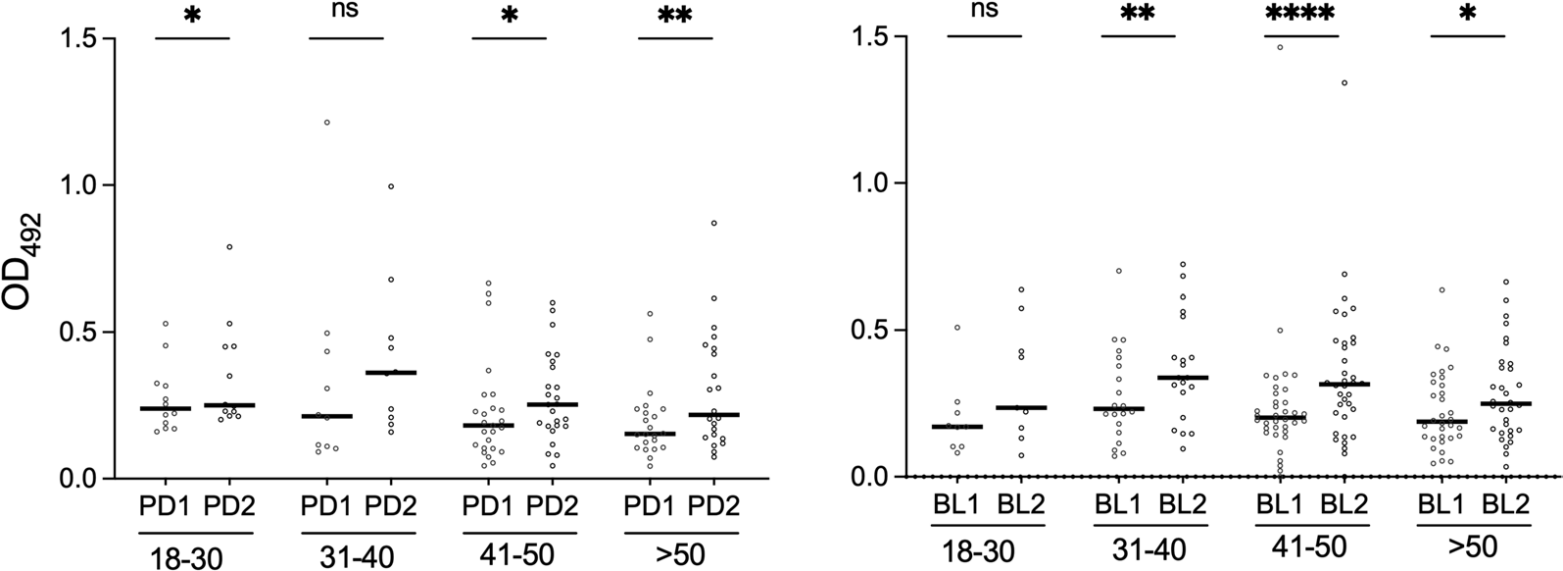
IgG responses to the *Ae. albopictus* alAg5-3 in different age groups. Pairwise comparisons of anti-alAg5-3 IgG responses in different age groups before (PD1, BL1) and after (PD2, BL2) the summer season of high mosquito density in Padua (left panel) and Belluno (right panel) are reported. The different age groups are shown at the bottom, with numbers indicating years. Only individuals sampled in both surveys (PD, *n* = 69; BL, *n* = 97) were included in the analysis. IgG levels are expressed as OD values. Dots mark individual values, and horizontal bars represent median values. Number of individuals in the different age groups as follows: PD (18–30, *n* = 12; 31–40, *n* = 10; 41–50, *n* = 25; > 50, *n* = 22), BL (18–30, *n* = 9; 31–40, *n* = 20; 41–50, *n* = 36; > 50, *n* = 32). Pairwise comparisons by the Wilcoxon matched pairs signed rank test

No decrease with age was found when participants were divided in four different age groups (18–30, 31–40, 41–50, and > 50 years old), with just one exception in the PD1 survey where a weakly significant difference was found between the 18–30 and the 41–50 years old groups (*P* = 0.0451, Supplementary Fig. S2). Importantly, when IgG responses to alAg5-3 were compared in individuals sampled in both surveys (PD, *n* = 69; BL, *n* = 97), a statistically significant increase after the summer season was found in most age groups in both study sites (Fig. 4). This observation indicates that variation of human exposure to *Ae. albopictus* can be effectively detected by the alAg5-3 antigen independently from age.

We also compared IgG responses to alAg5-3 in males and females. Since the Belluno cohorts had a strong sex-bias (9.2% and 12.2% females), we only considered the Padua surveys that were less unbalanced (30% and 25.8% females in PD1 and PD2, respectively). Although both median and mean values were always higher in females, this never reached statistical significance (Supplementary Fig. S3), and similar results were previously observed in the same groups with alSGE and al34k2 [58].

### IgG responses to a combination of alAg5-3 and al34k2

**Fig. 5:**
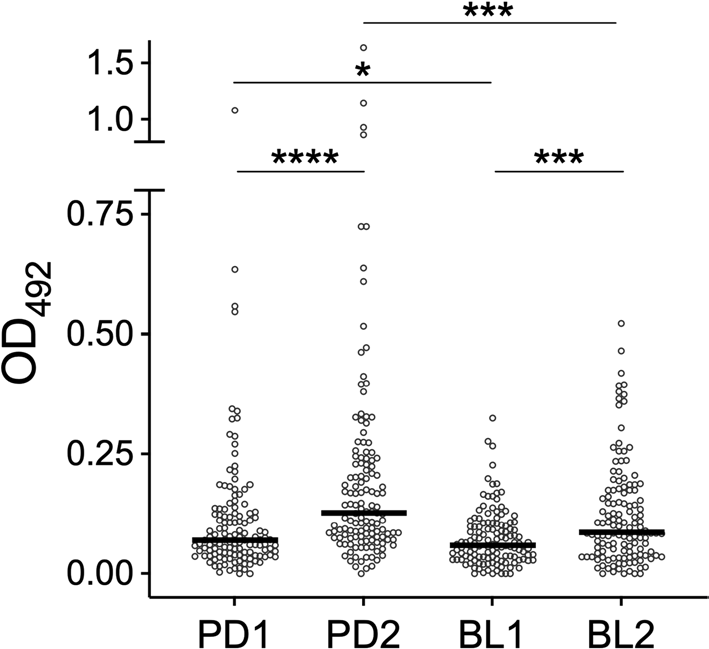
IgG responses to a combination of the *Ae. albopictus* alAg5-3 and al34k2 antigens. IgG levels against the two combined recombinant antigens in participants in the four different surveys (PD1, *n* = 130; PD2, *n* = 132; BL1, *n* = 130; BL2, *n* = 131). IgG levels are expressed as OD values. Dots mark individual values, and horizontal bars represent the medians. Pairwise comparisons by the Mann–Whitney *U* test

As recently reported by Chea and collaborators for the *Ae. aegypti* D7L1 and D7L2 salivary proteins, the combination of different mosquito salivary antigens may allow for the improvement of immunoassays for detecting variation of human exposure to mosquito vectors [56]. For this reason, a combination of both alAg5-3 and al34k2 was used to analyze the four cohorts from Padua and Belluno previously described. Similarly to what previously found with the alAg5-3 antigen alone, significantly higher IgG levels were found after the summer period of high mosquito density in both study sites (PD1 versus PD2, *P* < 0.0001; BL1 versus BL2, *P* < 0.001; Fig. 5). On the contrary, the use of both antigens allowed to reveal differences between the Padua and Belluno study sites (PD1 versus BL1, *P* < 0.05; PD2 versus BL2, *P* < 0.001) that were not detectable with alAg5-3 alone (Fig. 2).

We also verified whether IgG responses to the combination of both antigens showed any variation with age. Some slight but not substantial decrease with age was observed in Padua, both according to correlation analysis (PD2, *r* = −0.1889, *P* = 0.0301; Supplementary Table S2) and when age groups were compared (18–30 versus 41–50 years old, PD1, *P* = 0.0262, PD2, *P* = 0.0100), whereas no variation was found in Belluno. However, as observed with alAg5-3 alone, in individuals sampled in both surveys (PD, *n* = 69; BL, *n* = 97) IgG levels increased after summer in all age groups in both study sites, with the only exception of the > 50 years old group in Belluno.

## Discussion

Arboviral diseases transmitted by *Aedes* mosquitoes represent a serious threat for public health, as well exemplified by the 2024 outbreaks of dengue in the Americas region [30]. Moreover, several factors (global warming, insecticide resistance, spread of *Aedes* invasive species into temperate areas) point to a likely increase of arboviral diseases in endemic countries and to their possible spread into new ones. Vector monitoring and control still represent the main approaches to contain mosquito-borne viral diseases, and novel tools are urgently needed. In the context of vector control, the estimation of human–vector contact plays a crucial role, and the use of host antibody responses to vector salivary antigens can complement and overcome limitations of classical entomological measurements providing a more comprehensive toolbox for the assessment of human exposure to vector bites.

Toward the development of a sensitive marker of human exposure to *Ae. albopictus*, we have previously collected a set of sera from healthy individuals naturally exposed to mosquito bites in Padua and Belluno, and employed them to measure IgG responses: (i) to the *Ae. albopictus* 34k2 salivary protein (al34k2, a member of the 34 kDa family sharing around 33% amino acid identity with 34k1) [61, 79], and (ii) to salivary gland extracts (alSGE, which can be considered as a gold standard and used as a reliable reference) [58]. These same cohorts of individuals were used here to analyze IgG responses to alAg5-3, offering the opportunity for useful comparisons and considerations. Anti-alSGE IgG levels increased, after the summer season, in both study sites and were higher in Padua than Belluno in both surveys. This was perfectly concordant with the assumptions that during summer individuals are exposed to *Ae. albopictus* bites developing antisaliva antibodies, and with Padua being an area with higher tiger mosquito density than Belluno. A significant increase of anti-al34k2 IgG antibody levels was also found after the summer season in the cohorts from Padua, whereas statistical significance was achieved in Belluno only when the subset of individuals participating in both surveys (*n* = 97) was considered [58]. These observations suggested that anti-al34k2 IgG responses are suitable to detect seasonal variation of human exposure to *Ae. albopictus* but may not be sensitive enough in areas with low-to-moderate tiger mosquito density as Belluno. The alAg5-3 antigen analyzed here appeared more immunogenic than al34k2 in a pilot study (Fig. 1) and, in addition, anti-alAg5-3 IgG levels were significantly higher after summer exposure in both study sites, Padua and Belluno (Fig. 2). These findings clearly point to IgG responses to alAg5-3 as a more sensitive marker to evaluate temporal variation of human exposure to *Ae. albopictus*, suggesting that its use may be especially recommended in areas where *Ae. albopictus* density is low or moderate.

Antibody responses to mosquito salivary antigens can be useful not only to detect seasonal variation of exposure to vector bites, but also to reveal differences in human–vector contact and disease risk between two or more distinct areas. Our two study sites were selected as districts with “high” (Padua) and “low-to-moderate” (Belluno) exposure to tiger mosquito bites according to the following considerations. First, the difference in time of colonization and establishment in the two sites; in fact, *Ae. albopictus* was first reported in Padua around 1990 [64] and in Belluno more than 20 years later [65], which implies that individuals from these two locations have a different history of exposure to bites from this mosquito species. Second, the different *Ae. albopictus* density in the two areas, as indicated by published entomological data [68] as well as by the annual entomological monitoring performed by one of the coauthors in the years preceding sera collection (F. Montarsi, unpublished observations; for additional details see [58]). Entomological surveys conducted during the sera collection periods showed differences between the study sites that were not so marked as expected from previous observations (Supplementary Table S1). Nevertheless, higher anti-alSGE and anti-al34k2 IgG levels were found in Padua as compared with Belluno in both the first and second survey, making us confident in the difference in *Ae albopictus* density between the two areas [58]. On the contrary, anti-alAg5-3 IgG levels were very similar in the two study sites or even slightly higher in Belluno (Fig. 2). We do not have a sound and clear-cut explanation for this lack of site-specific differences with alAg5-3, which may reflect multiple factors as for example the higher immunogenicity of alAg5-3, the different history of exposure to *Ae. albopictus*, and local vector species composition. In this respect, one difference between the two study sites is that in Padua *Ae. albopictus* is the only or very largely predominant *Aedes* species, whereas in the Belluno area *Ae. koreicus* is also present, although with a significantly lower prevalence in comparison to the tiger mosquito (9.7% versus 57% in the years immediately preceding sera collection) [66–68]. Taking advantage of the recently released *Ae. koreicus* genome [72], we used tblastn [80] to search the reference genome assembly Akor_1.1 using al34k2 and alAg5-3 as queries. Putative *Ae. koreicus* orthologous proteins (koAg5-3 and ko34k2) were identified, with the two Ag5-3 mature proteins being more closely related (81% identity) than 34k2 proteins (64% identity, Supplementary Figs. S4 and S5). This quite different degree of conservation suggests a possible explanation of why site-variation was observed when comparing anti-al34k2 IgG levels whereas no difference was found with the alAg5-3 antigen. In fact, owing to the higher amino acid identity, anti-alAg5-3 IgG responses measured in Belluno may reflect the cumulative exposure to *Ae. albopictus* and *Ae. koreicus*; on the contrary, the lower conservation of the 34k2 protein between the two species may imply a limited or absent cross-reactivity and higher species-specificity. Of note, indications of low cross-reactivity has been previously reported between al34k2 and the *Ae. aegypti* ortholog ae34k2; in fact, despite a high conservation (62% identity), no cross-reactivity was observed in a murine model [60] and low cross-reactivity was observed in a naturally exposed population from Bolivia [59]. These speculations, which will need further experimental verification, may have relevant implications and raise questions regarding the extent of cross-reactivity and degree of specificity of the alAg5-3 antigen (species- versus genus-specificity).

We also verified whether using a mixture of the two antigens, alAg5-3 and al34k2, could provide any advancement to sensitivity and/or specificity of our immunoassay. This was justified by the previously reported intrinsic inter-individual variability of IgG responses to mosquito salivary proteins, with some individuals exhibiting an IgG antibody response against a given antigen and no response to a different one from the same species, or vice versa [81]. Moreover, Chea and collaborators have recently reported that a mix of the *Ae. aegypti* D7L1 (aeD7L1) and D7L2 (aeD7L2) proteins was a better marker of exposure to the yellow fever mosquito than each antigen alone [56]. We found that IgG responses to alAg5-3 plus al34k2 allowed us to reveal both temporal and spatial variation of exposure to the tiger mosquito, as indicated by the higher IgG levels found (i) after summer exposure in both sites and (ii) in Padua as compared with Belluno both before and after the summer period (Fig. 5). Intriguingly, median OD levels were lower in all surveys in comparison with those obtained using alAg5-3 alone (see Figs. 2 and 5), and very similar results were obtained in a previous study on the aeD7L1 and aeD7L2 proteins [56]. The reason(s) for these observations are unclear (perhaps protein–protein interactions, which may be also favored by the increase in total protein amount, may result in some form of epitope masking) but we cannot provide any data-supported explanation for this decrease. However, the combination of the two antigens better reproduced what was previously observed using the *Ae. albopictus* salivary gland extracts (alSGE), which can be reliably considered as a reference gold standard.

It is known that long-term natural exposure to mosquito bites may induce a decrease of both human cutaneous reactions and antibody responses to mosquito saliva over time, possibly owing to the development of immune tolerance and progressive desensitization to mosquito salivary proteins [33, 74, 75, 77, 78]. However, the evolution with age of IgG responses to individual salivary proteins may depend on several factors and appears to be antigen specific. For example, in the same group of individuals from Burkina Faso, we previously reported that IgG responses to the *An. gambiae* salivary proteins gSG6 and cE5 decreased and increased with age, respectively [81]. This age-dependence of IgG responses to salivary antigens should be taken into consideration in the context of assessment of exposure to mosquito bites, because unbalanced age distributions may represent a source of bias. IgG responses to alSGE and al34k2 were found to significantly decrease with age in Padua but not in Belluno, perhaps because of the different intensity of bites and history of colonization by the tiger mosquito between the two sites [58]. A weakly significant decrease with age was only found in Padua with alAg5-3 (Supplementary Figs. 3, S1 and S2), suggesting that IgG responses to alAg5-3 are not significantly influenced by age (Fig. 4). We cannot provide any concrete evidence to explain the less age-dependent IgG responses to alAg5-3 as compared with al34k2; perhaps an analysis of IgG subclasses may help shed some light on this issue. In any case, the limited influence of age on IgG responses to alAg5-3 should be taken into consideration and may represent an additional advantage.

Compiled according to *P* values determined by pairwise comparisons (seasonal and site variation, Mann Whitney *U* test) and by correlation analysis (age effect, Spearman correlation). Seasonal variation refers to the comparisons before/after summer exposure in each study site. Site variation indicates the comparisons between the study sites both before and after the summer period. Age effects, that is the propensity of IgG responses to decrease with age, is reported for the two study sites Padua (PD = PD1 + PD2, *n* = 262) and Belluno (BL = BL1 + BL2, *n* = 261). Additional info on Spearman correlation analysis in the four surveys can be found in the Supplementary Table S2. *P* values as indicated in the Methods section. Data on alSGE and al34k2 from Buezo Montero et al. [58].

Overall, IgG responses to alAg5-3 appeared to be a sensitive marker of human exposure to *Ae. albopictus*. The higher immunogenicity of alAg5-3 in comparison with al34k2 makes its use recommended for longitudinal studies (as in Padua or in Belluno here), especially in conditions of low or moderate tiger mosquito density. This is certainly true for areas where *Ae. albopictus* is the only or largely predominant *Aedes* species; however, local vector species composition should be carefully considered in view of the high conservation of the Ag5-3 salivary protein among *Aedes* species, which raises questions regarding cross-reactivity and species-specificity. Notably, the *Ae. aegypti* protein aeAg5-3 (UniProt A0A1S4F3J3) shares very high amino acid identity (83%) with alAg5-3. In this scenario, it is possible that IgG responses to alAg5-3 may represent a sensitive genus-specific marker to detect exposure to *Aedes* mosquitoes species, although experimental data will be needed to confirm this possibility. IgG responses to alAg5-3 did not appear instead suitable to reveal variation of exposure between Padua and Belluno, perhaps because of local vector species composition and cross-reactivity, as mentioned above. Therefore, the use of al34k2, which appeared to have a significantly higher species-specificity, would be so far the most appropriate choice for cross-sectional studies, when human exposure to the tiger mosquito may need to be compared in different areas (Table 1). The use of a mixture of alAg5-3 and al34k2 should also be considered since (i) it may be useful for both longitudinal and cross-sectional studies and (ii) appeared to better recapitulate IgG responses to *Ae. albopictus* salivary extracts (Table 1); however, further analyses in different epidemiological settings will be needed to fully understand the possible benefits of the combined use of these two antigens. As far as the other and most relevant arboviral disease vector is concerned, at the moment the best available markers of human exposure to *Ae. aegypti* appear to be aeD7L1 and aeD7L2 [56]. They share 69% and 72% identity with the *Ae. albopictus* orthologs and, therefore, may be able to reveal exposure to the tiger mosquito. The use of sera from individuals exposed to *Ae. aegypti* but not to *Ae. albopictus*, and vice versa, is expected to clarify the degree of species- and/or genus-specificity of the Ag5-3, D7L1 and D7L2 proteins. Overall, these candidates hold great promises toward the development of genus- and/or species-specific markers of human exposure to these two important arboviral disease vectors. These additional serological tools are expected to be of great help both for epidemiological studies and transmission risk assessment, as well as for the evaluation of efficacy of classic (insecticides-based) or more innovative (sterile insect technique, population suppression) strategies for vector control interventions. A further step toward the optimization and standardization of tools for the assessment of human–vector contact may involve the switch from recombinant salivary protein to synthetic peptides conserving sufficient immunogenicity as compared with the native proteins, which may allow to improve the reproducibility and feasibility of the immunoassay.

## Conclusion

In conclusion, in this study we tested a novel candidate marker of human exposure to *Ae. albopictus* (alAg5-3) and had the opportunity to compare the responses of the same groups of individuals with different salivary antigens from the tiger mosquito (alSGE, al34k2, alAg5-3, and alAg5-3 + al34k2). A tentative summary of the sensitivity in revealing seasonal and site variation of exposure exhibited by these different antigens, as well as of the possible influence of age on the antigen-specific IgG responses, is presented in Table 1.

**Table 1:** Summary table comparing the performance of the different antigens

Antigen	Seasonal variation		Site variation		Age effect	
	PD1 versus PD2	BL1 versus BL2	PD1 versus BL1	PD2 versus BL2	PD	BL
alSGE	****	***	*	**	****	ns
al34k2	**	ns	****	****	***	*
alAg5-3	****	****	ns	ns	*	ns
alAg5-3 + al34k2	****	***	*	***	ns	ns

## Supplementary Information

Additional file 1.

## Data Availability

Data generated during this study are included in this published article (and its supplementary information files).
