# Supplementary material for: A novel biomarker of human exposure to Aedes albopictus based on the Ag5-3 salivary protein from the tiger mosquito
Source: Parasit Vectors. 2025 Nov 19;18:470. doi: 10.1186/s13071-025-07118-x (PMC12628915; doi:10.1186/s13071-025-07118-x)
Supplement: Supplementary file 1 — Additional file 1. [file 13071_2025_7118_MOESM1_ESM.docx]

**Supplementary Figures and Tables**

**A novel biomarker of human exposure to *Aedes albopictus* based on the Ag5-3 salivary protein from the tiger mosquito.**

**Maria Greta Dipaola^1†^, Eleonora Perugini^1†^, Giulia Mancini^2^, Nicolò Gennari^2^, Paola Serini^1^, Giulia Bevivino^1^, Alessio Borean^3^, Fabrizio Lombardo^1^, Marco Pombi^1^, Fabrizio Montarsi^4^, Paolo Gabrieli^5^, Federico Forneris^2^, Bruno Arcà^1*^**

† Maria Greta Dipaola and Eleonora Perugini contributed equally to this work.

1. *Department of Public Health and Infectious Diseases, Sapienza University of Rome, Rome, Italy.*
2. *Department of Biology and Biotechnology “L. Spallanzani”, University of Pavia, Pavia, Italy.*
3. *Department of Transfusion Medicine, San Martino Hospital, Belluno, Italy.*
4. *Istituto Zooprofilattico Sperimentale delle Venezie, Legnaro, Italy.*
5. *Department of Biosciences, University of Milan, Milan, Italy.*

*****Correspondence: [bruno.arca@uniroma1.it](mailto:bruno.arca@uniroma1.it)

**
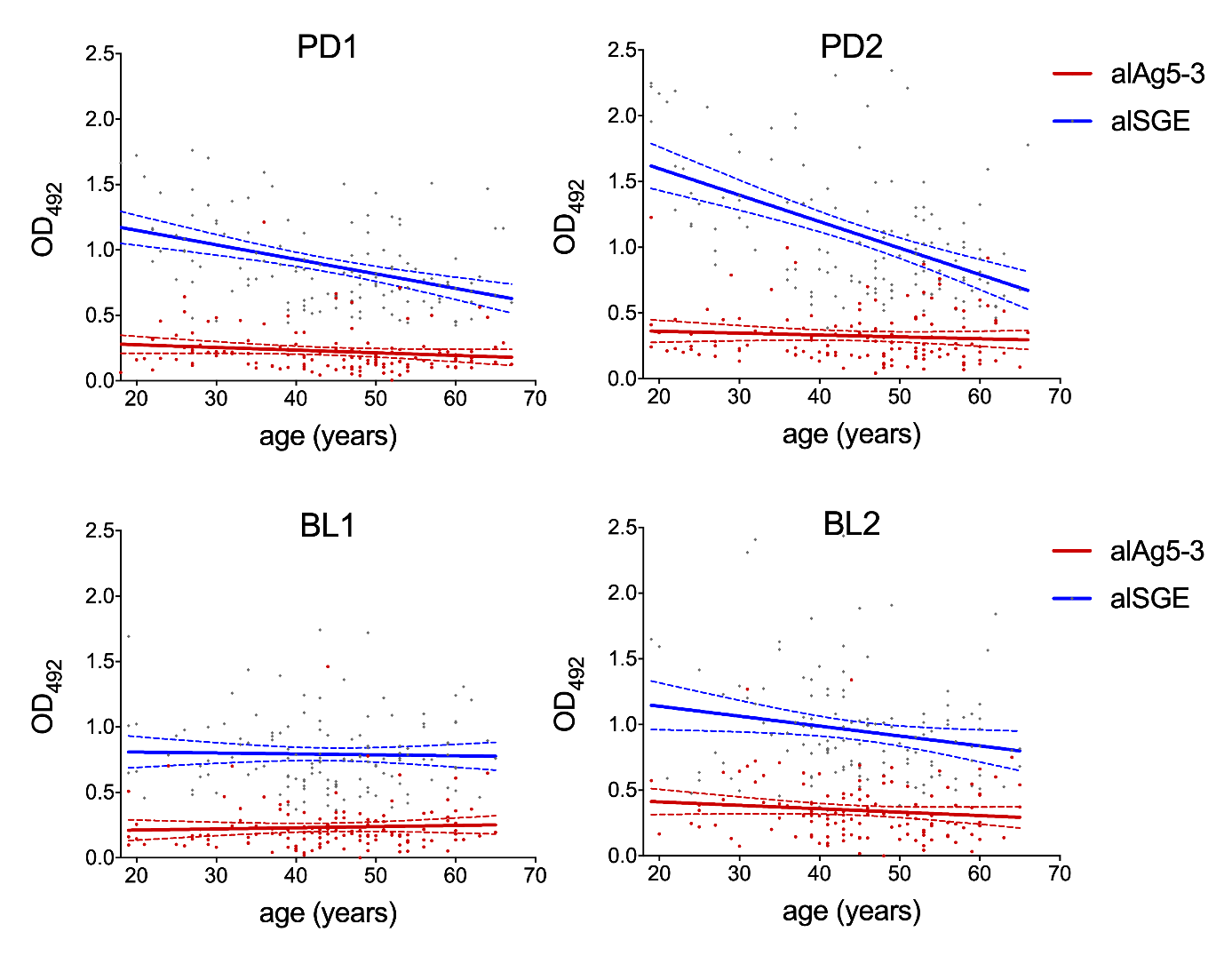
**

**Figure S1. IgG responses to the *Ae. albopictus* alAg5-3 according to age in the four different surveys.** Scatter plot of IgG responses to alAg5-3 (red) as function of age in Padua (PD1, n=130; PD2, n=132) and Belluno (BL1, n=130; BL2, n=131). Responses to alSGE (blue) in the same groups of individuals as determined in a previous study [1] are shown for comparison. IgG levels are expressed as OD values and dots mark individual values. Best-fit lines (solid lines) and confidence interval bands (dashed lines) are shown. Spearman correlation: PD1-alAg5-3 (r = -0.1870, p=0.0338), PD1-alSGE (r = -0.3832, p<0.0001), PD2-alAg5-3 (r = -0.0779, p=0.3746), PD2-alSGE (r = -0.4570, p<0.0001), BL1-alAg5-3 (r = 0.0814, p=0.3575), BL1-alSGE (r = 0.0007, p=0.9933), BL2-alAg5-3 (r = -0.1347, p=0.1250), BL2-alSGE (r = -0.1648, p=0.0600).

**
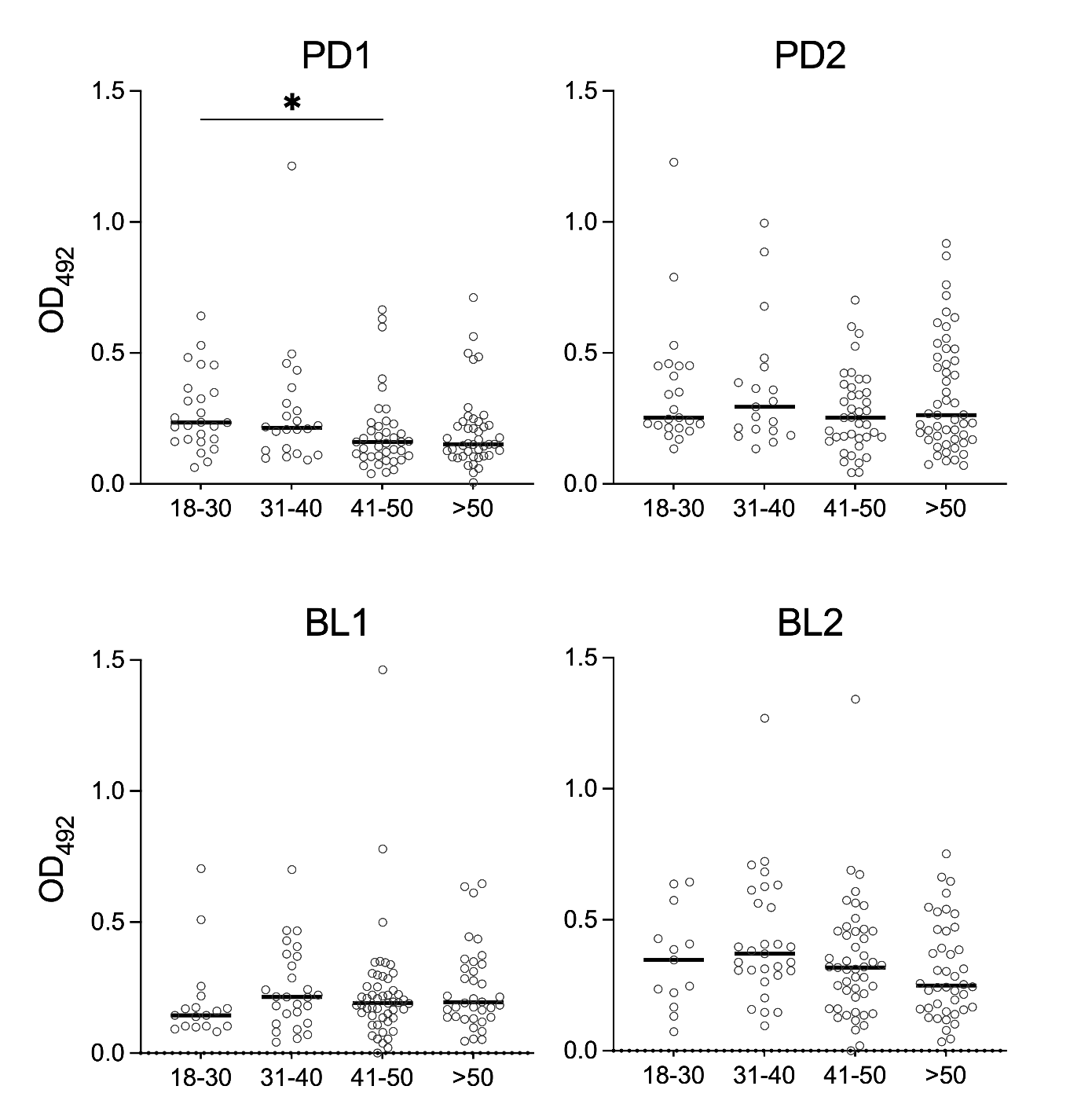
**

**Figure S2. IgG responses to the *Ae. albopictus* alAg5-3 in the four different surveys according to age groups.** Anti-alAg5-3 IgG responses in the four different age groups as indicated at the bottom (18-30, 31-40, 41-50, >50 years old) are reported. The different surveys are shown at the top of each graph. IgG levels are expressed as OD values. Dots mark individual values, and horizontal bars represent the medians. Number of individuals in the different age groups as follows: PD1 (18-30, n=25; 31-40, n=22; 41-50, n=39; >50, n=43), PD2 (18-30, n=23; 31-40, n=19; 41-50, n=39; >50, n=51), BL1 (18-30, n=17; 31-40, n=27; 41-50, n=50; >50, n=36), BL2 (18-30, n=13; 31-40, n=29; 41-50, n=47; >50, n=42). Kruskal-Wallis with Dunn’s multiple comparisons test.

**
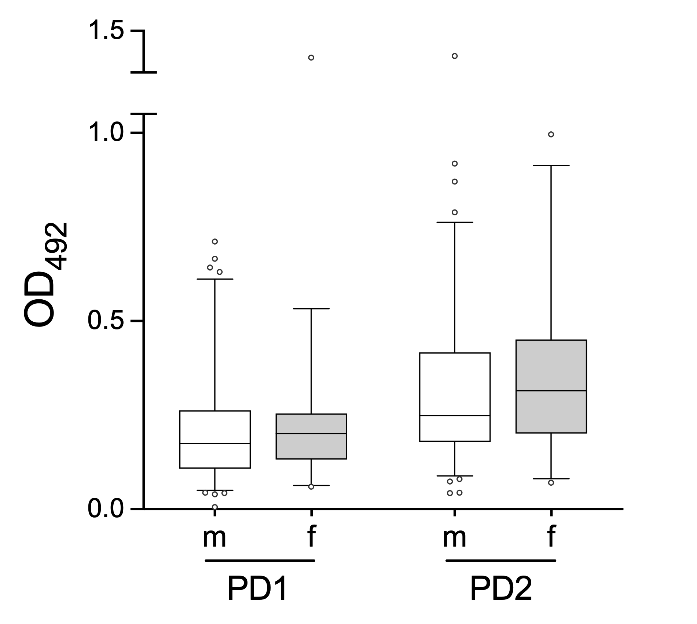
**

**Figure S3. IgG responses to the *Ae. albopictus* alAg5-3 in males and females.** Anti-alAg5-3 IgG levels in males (m) and females (f) from the Padua study site (PD1, f=39, m=91; PD2, f=34, m=98). IgG levels are expressed as OD values. Boxes display median OD values, 25th and 75th percentiles; whiskers represent 5th and 95th percentiles and dots the outliers. In all cases pairwise comparisons within each survey showed no significant difference (Mann-Whitney U test, p value >0.05).


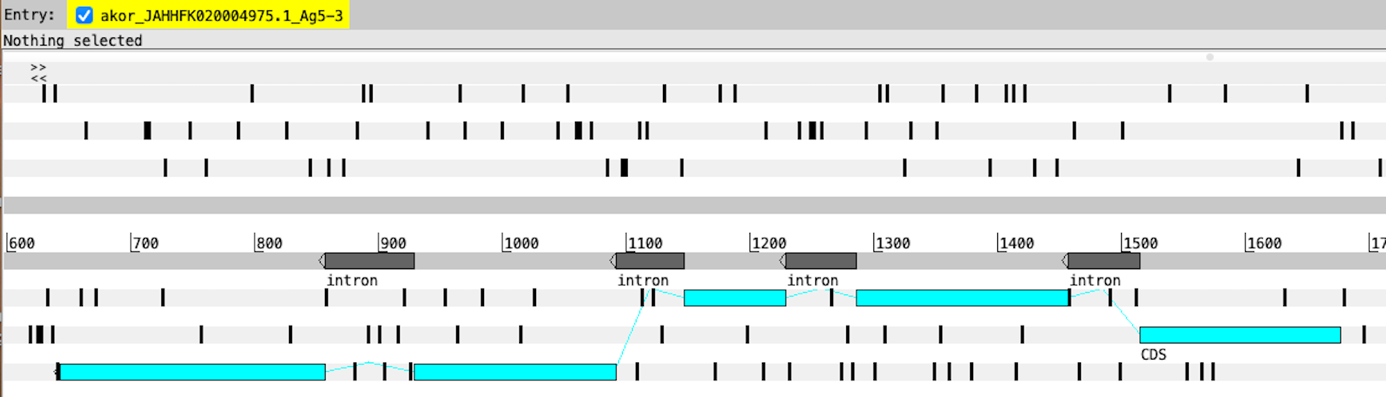


>akor_Ag5-3_gene (akor_1.1_JAHHFK020004975.1)

ATGGGCGTTTTTCCAACTCGGAGCAATTATGTATTATTCGCAGTAGTTGTCGTCTTGTTGCCAGTGGCGTCTGTGCTCGGTGCCGGAAAAGACTATTGCGGTAGCACATATAAACGCTTGTGTGAAAACAAGGGCGATCATGTTGGATGTCGCCCAAAAGAC**gtaagaacgatttttaaatttttgaagcaccaagaaatcgtatattttgattttatag**TTTTCGGATTATCCTTCGTGCAGTGGTCAACATCCTAAAATGATCAAAGTTACCTCGAAGTATCAAAGGCAAATCATGAATCTCCACAACCAACTGAGGGCTAAACTAGCAAGTGGGAAAATGTCTTCAACTTATGGAACTTTTCCTAGTGCTATGAACATGTCAGAACTT**gtaagtaacgtagttgtttgaagacatttatctttatcacagtttttattgttgaag**AAATGGGACAACGAGTTAGCCAAGCTTGCTGAATACAATGTCAAGCAGTGTACTATGAACCACGATCGCTGCAGGAGTACAG**gttatacagtttggtttagtgcatgaacctcataactaaaagcaacttattacag**CAAAATTCAAGGACGCTGGTCAAAATATTTACTATTCGTCATGGTCTCAAAAAAGGTCCAAGGATAAGACAAAACTAATTGCAGAAGCCATTCAAGCTTGGTGGGATGAGCACAAGGATTTCTACTTAAACGAGGTTGACCGGTTCGATGGTCAAAGCAGAGG**gtaagatagattgaaagcttgttgaataactttcctaacttcattttaatcgccacctacgttttgttttag**TGTTTTGCATTTCACCGCGATGGCTGTAGATTACCAGACCCACGTCGGATGCGCCATATCAGAATACGATTACGCCGGAACTGGAGATACTTTTCTCATGACGTGCAACTATTCGTCGTGGACATGGCTTAGCCAACCAATCTATCAAAAAGGGAGATCCTGTTCCAAATGTTCGAAAAAATGTAGCACGACCTATAAATCTCTGTGCAGTGCTTGA

>akor_Ag5-3_pep

MGVFPTRSNYVLFAVVVVLLPVASVLGAGKDYCGSTYKRLCENKGDHVGCRPKDFSDYPSCSGQHPKMIKVTSKYQRQIMNLHNQLRAKLASGKMSSTYGTFPSAMNMSELKWDNELAKLAEYNVKQCTMNHDRCRSTAKFKDAGQNIYYSSWSQKRSKDKTKLIAEAIQAWWDEHKDFYLNEVDRFDGQSRGVLHFTAMAVDYQTHVGCAISEYDYAGTGDTFLMTCNYSSWTWLSQPIYQKGRSCSKCSKKCSTTYKSLCSA

|  | Ae. albopictus | Ae. aegypti | Ae. koreicus |
| --- | --- | --- | --- |
| Ae. albopictus | 100 |  |  |
| Ae. aegypti | 83% (89%) | 100 |  |
| Ae. koreicus | 81% (90%) | 79% (88%) | 100 |

**Figure S4. The *Aedes koreicus* putative orthologue of alAg5-3.** The five exon structure of the putative *Ae. koreicus Ag5-3* gene as reconstructed by the Artemis tool is shown on the top (exons in light blue, introns in grey). The coding sequence from ATG to stop codon (with introns in bold and lowercase), and the amino acid sequence of the putative koAg5-3 protein follow (signal peptide as predicted by SignalP-5.0 website at https://services.healthtech.dtu.dk/services/SignalP-5.0/ highlighted in yellow). The table at the bottom reports the percentages of identity and similarity (in brackets) between the Ag5-3 salivary proteins of *Ae. albopictus*, *Ae. aegypti* and *Ae. koreicus* as determined by blastp alignment of the mature proteins.


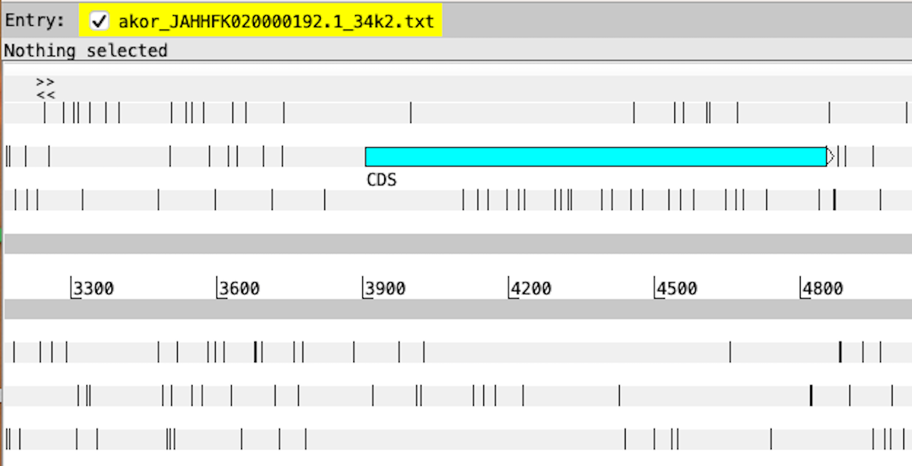


>akor_34k2_cds (akor_1.1_JAHHFK020000192.1)

ATGGAACCCATTCTCACGTTCGCTTTCCTCGTGGCAATCCTCCTTCCGGCCGGCCATCCCAATCCACTACCCTCGAAGGAATGCACCGCCACTGAGGACGATCTCAGCTCGATCAAATCAGCCATACAAAGAGCCACCGGAGGCAGGGCGTCGCCGGATGGCATTCTCCCCCAGGAAACCCTCGACCGGTGTCCAATGTTGAAGGCCATCGCCGGGAAAATCAAGTCCGTAGCCGAAGAAATCATCCACCTGAAGGAAAGCTCCATCACGACCGAGCAGTTGGACGAGCTGAAGGAATCGTTCGAGCAGAAGGTGAACGAAATCATGAAAAGTCGGGATATTTTCGAGAAGGAATCCAATCTCGACGCGACCAAGGAGCGCGGGCAGATGATCGATCGATTGACGGCCCTCCAGGTGAAGGTGACGGAACTGGAGAAGGAAATCGAGGAGAAGACAAAGCAAACGTACGAGAACATGGCCGAGTTGATTTTCGAGCGGCTGCAGATGAACAGTACGGACCTCATTCGGAACTACACGAAGCGCATGATGCATGAGAAGATGGACGAACTGATGCGCAAGCTGGAAACGGATTACAGAATTTTCCTCGGTGCGTTGCGATTCCTGAATCACCTGGATGATCAGAATCTGATCGATAAGGTGTTCGACGGGATTTTGACGCGACTGGACGAGATGCCGCTGGATAACGATAAGGAACGCGAGAGAGGAAAGTACGTCCTGGTGAATCTGCTATGCTGGACGGTGAATAACGCATTTCTGACGAAGAAGTACGTCCAAAAGAAGACCGAATTGTTCCGGATTGCGTTGAAGTTCTATCCCAACACCGGCAACACAGAGGCCAACGCCGCGGATGTTCGGAGCCGGCAGTATTGCGATGCCAAGTTCCCGGCCAATGTCATCACGTGGTTTGCAGTGAGCCATGGGAAGTAG

>akor_34k2_pep

MEPILTFAFLVAILLPAGHPNPLPSKECTATEDDLSSIKSAIQRATGGRASPDGILPQETLDRCPMLKAIAGKIKSVAEEIIHLKESSITTEQLDELKESFEQKVNEIMKSRDIFEKESNLDATKERGQMIDRLTALQVKVTELEKEIEEKTKQTYENMAELIFERLQMNSTDLIRNYTKRMMHEKMDELMRKLETDYRIFLGALRFLNHLDDQNLIDKVFDGILTRLDEMPLDNDKERERGKYVLVNLLCWTVNNAFLTKKYVQKKTELFRIALKFYPNTGNTEANAADVRSRQYCDAKFPANVITWFAVSHGK

|  | Ae. albopictus | Ae. aegypti | Ae. koreicus |
| --- | --- | --- | --- |
| Ae. albopictus | 100 |  |  |
| Ae. aegypti | 63% (82%) | 100 |  |
| Ae. koreicus | 64% (81%) | 61% (81%) | 100 |

**Figure S5. The *Aedes koreicus* putative orthologue of al34k2.** The intronless structure of the putative *Ae. koreicus 34k2* gene as reconstructed by the Artemis tool is shown on the top. The coding sequence from ATG to stop codon, and the amino acid sequence of the putative ko34k2 protein follow (signal peptide as predicted by SignalP-5.0 website highlighted in yellow). The table at the bottom reports the percentages of identity and similarity (in brackets) between the 34k2 salivary proteins of *Ae. albopictus*, *Ae. aegypti* and *Ae. koreicus* as determined by blastp alignment of the mature proteins.

**Table S1. Entomological monitoring in Padua and Belluno during the study period**

| **PADUA** | | | | **BELLUNO** | | | |
| --- | --- | --- | --- | --- | --- | --- | --- |
| **Date** | **Eggs** | **Eggs/Trap** | **% Ovitraps** | **Date** | **Eggs** | **Eggs/Trap** | **% Ovitraps** |
| May 26 | 219 | 19.9 | 34.3 | May 23 | 0 | 0 | 0 |
| June 9 | 1011 | 63.2 | 48.5 | June 7 | 218 | 36.3 | 30 |
| June 23 | 1046 | 41.8 | 67.6 | June 20 | 2618 | 137.8 | 95 |
| July 7 | 3843 | 120.1 | 97 | July 7 | 3635 | 201.9 | 90 |
| **Period I** | 6119 | 72.8 | 62.2 | **Period I** | 6471 | 150.5 | 53.75 |
|  |  |  |  |  |  |  |  |
| Aug 25 | 9848 | 281.4 | 94.6 | Aug 23 | 2280 | 114 | 100 |
| Sept 7 | 6473 | 215.8 | 96.8 | Sept 5 | 5474 | 342.1 | 88.9 |
| Sept 22 | 3104 | 107 | 78.4 | Sept 22 | 1391 | 86.9 | 84.2 |
| Oct 6 | 1698 | 53.1 | 91.4 | Oct 3 | 421 | 38.3 | 55 |
| **Period II** | 21123 | 167 | 90 | **Period II** | 9566 | 151.8 | 81.8 |

Results of the entomological surveys carried out using oviposition standard traps in the two study areas, Padua and Belluno. Monitoring was performed, in parallel to sera collection, at the indicated dates during the periods May-July (Period I) and August-October 2017 (Period II). Total number of eggs, mean number of eggs per positive ovitraps and percentange of positive ovitraps are reported. Data from Buezo Montero *et al.* [1], where additional details including a map with ovitraps location in the two study sites can be found (see supplementary material downloadable at <https://www.frontiersin.org/journals/cellular-and-infection-microbiology/articles/10.3389/fcimb.2020.00377/full#supplementary-material>).

**Table S2. Correlation between age and IgG levels in the two study sites Padua and Belluno**

| survey | antigen | Spearman r | 95% CI | p-value summary | p-value |
| --- | --- | --- | --- | --- | --- |
| **PD1** (n=130) | alSGE | -0.3832 | -0.53 to -0.22 | **** | <0.0001 |
|  | al34k2 | -0.2551 | -0.41 to -0.08 | ** | 0.0035 |
|  | alAg5-3 | -0.1870 | -0.35 to -0.01 | * | 0.0338 |
|  | alAg5-3 + al34k2 | -0.0792 | -0.25 to 0.10 | ns | 0.3723 |
| **PD2**  (n=132) | alSGE | -0.4570 | -0.59 to -0.31 | **** | <0.0001 |
|  | al34k2 | -0.2435 | -0.40 to -0.07 | ** | 0.0049 |
|  | alAg5-3 | -0.0779 | -0.25 to 0.10 | ns | 0.3746 |
|  | alAg5-3 + al34k2 | -0.1889 | -0.35 to -0.01 | * | 0.0301 |
| **PD** (n=262) | alSGE | -0.3956 | -0.50 to -0.28 | **** | <0.0001 |
|  | al34k2 | -0.2190 | -0.33 to -0.10 | *** | 0.0004 |
|  | alAg5-3 | -0.1225 | -0.24 to -0.003 | * | 0.0481 |
|  | alAg5-3 + al34k2 | -0.0952 | -0.22 to -0.03 | ns | 0.1248 |
| **BL1** (n=130) | alSGE | 0.0007 | -0.18 to 0.18 | ns | 0.9933 |
|  | al34k2 | -0.0961 | -0.27 to 0.08 | ns | 0.2769 |
|  | alAg5-3 | 0.0814 | -0.01 to 0.25 | ns | 0.3575 |
|  | alAg5-3 + al34k2 | 0.0696 | -0.11 to 0.24 | ns | 0.4312 |
| **BL2** (n=131) | alSGE | -0.1648 | -0.33 to 0.01 | ns | 0.0600 |
|  | al34k2 | -0.2149 | -0.38 to 0.04 | * | 0.0137 |
|  | alAg5-3 | -0.1347 | -0.30 to 0.04 | ns | 0.1250 |
|  | alAg5-3 + al34k2 | -0.1679 | -0.33 to 0.009 | ns | 0.0552 |
| **BL** (n=261) | alSGE | -0.0715 | -0.19 to 0.05 | ns | 0.2495 |
|  | al34k2 | -0.1503 | -0.27 to -0.03 | * | 0.0151 |
|  | alAg5-3 | -0.0207 | -0.15 to 0.10 | ns | 0.7389 |
|  | alAg5-3 + al34k2 | -0.0551 | -0.18 to 0.07 | ns | 0.3749 |

Variation of individual IgG responses to salivary antigens according to age in the different surveys (PD1, PD2, BL1, BL2) and study sites (PD=PD1+PD2; BL=BL1+BL2) according to Spearman correlation analysis. Number of individuals, Spearman correlation coefficients, 95% Confidence Interval and p-values are reported. P values as indicated in the Methods section. Data on alSGE and al34k2 from Buezo Montero *et al.* [1].

1. Buezo Montero S, Gabrieli P, Montarsi F, Borean A, Capelli S, De Silvestro G, et al. IgG Antibody Responses to the Aedes albopictus 34k2 Salivary Protein as Novel Candidate Marker of Human Exposure to the Tiger Mosquito. Front Cell Infect Microbiol. 2020;10:377; doi: 10.3389/fcimb.2020.00377. <https://www.ncbi.nlm.nih.gov/pubmed/32850479>.
